# Contrasting patterns of selection between MHC I and II across populations of Humboldt and Magellanic penguins

**DOI:** 10.1002/ece3.2502

**Published:** 2016-09-28

**Authors:** Nicole Sallaberry‐Pincheira, Daniel González‐Acuña, Pamela Padilla, Gisele P. M. Dantas, Guillermo Luna‐Jorquera, Esteban Frere, Armando Valdés‐Velásquez, Juliana A. Vianna

**Affiliations:** ^1^ Laboratorio de Biodiversidad Molecular Departamento de Ecosistemas y Medio Ambiente Facultad de Agronomía e Ingeniería Forestal Pontificia Universidad Católica de Chile Santiago Chile; ^2^ Escuela de Medicina Veterinaria Facultad Ecología y Recursos Naturales Universidad Andrés Bello Santiago Chile; ^3^ Facultad de Ciencias Veterinarias Universidad de Concepción Chillán Chile; ^4^ Pontificia Universidade Catolica de Minas Gerais Belo Horizonte Brazil; ^5^ Universidad Católica del Norte Millenium Nucleus of Ecology and Sustainable Management of Oceanic Islands ESMOI Centro de Estudios Avanzados en Zonas Áridas CEAZA Coquimbo Chile; ^6^ Centro de Investigaciones de Puerto Deseado Universidad Nacional de la Patagonia Austral Puerto Deseado Argentina; ^7^ Laboratorio de Estudios en Biodiversidad Facultad de Ciencias Biológicas y Fisiológicas Universidad Peruana Cayetano Heredia Lima Peru

**Keywords:** Adaptation, MHC, positive selection, *Spheniscus*, trans‐species alleles

## Abstract

The evolutionary and adaptive potential of populations or species facing an emerging infectious disease depends on their genetic diversity in genes, such as the major histocompatibility complex (MHC). In birds, MHC class I deals predominantly with intracellular infections (e.g., viruses) and MHC class II with extracellular infections (e.g., bacteria). Therefore, patterns of MHC I and II diversity may differ between species and across populations of species depending on the relative effect of local and global environmental selective pressures, genetic drift, and gene flow. We hypothesize that high gene flow among populations of Humboldt and Magellanic penguins limits local adaptation in MHC I and MHC II, and signatures of selection differ between markers, locations, and species. We evaluated the MHC I and II diversity using 454 next‐generation sequencing of 100 Humboldt and 75 Magellanic penguins from seven different breeding colonies. Higher genetic diversity was observed in MHC I than MHC II for both species, explained by more than one MHC I loci identified. Large population sizes, high gene flow, and/or similar selection pressures maintain diversity but limit local adaptation in MHC I. A pattern of isolation by distance was observed for MHC II for Humboldt penguin suggesting local adaptation, mainly on the northernmost studied locality. Furthermore, trans‐species alleles were found due to a recent speciation for the genus or convergent evolution. High MHC I and MHC II gene diversity described is extremely advantageous for the long‐term survival of the species.

## 1 Introduction

Phylogeographical patterns have been evaluated using neutral markers, primarily analyzing mitochondrial DNA and specific nuclear introns or microsatellites. However, neutral markers do not reflect adaptive genetic diversity, which is the primary factor that allows for evolutionary change and local adaptation, because these markers are not subject to selection (Reed & Frankham, 2001). Therefore, adaptive markers are required to understand the distribution of this level of genetic variation (Marsden et al., 2012). Furthermore, low genetic variation can reduce long‐term population persistence in the wild due to a limited ability to respond to environmental change and/or novel pathogens. In addition, inbreeding increases the expression of recessive deleterious alleles and/or the loss of heterozygote advantage, causing a decline in fitness (Allendorf & Luikart, 2007; Taylor, Jenkins, & Arcese, 2012).

An adaptive marker used to evaluate selection driven by diseases is the major histocompatibility complex (MHC) which includes multiple genes involved in the immune response (Piertney & Oliver, 2006). Therefore, evolutionary and adaptive potentials of endangered populations and species relative to the threat of an emerging disease have been studied assessing MHC (e.g., Bollmer, Vargas, & Parker, 2007; Garrigan & Hedrick, 2003; Yuhki & O'Brien, 1990). The MHC is a gene family related to species resistance to pathogens, and is a key element of the vertebrate immune system, responsible for the presentation of foreign peptides to lymphocyte T cells (Klein, 1986). MHC genes are the most polymorphic genes described in vertebrates, which encode a set of transmembrane proteins critical to the generation of immune responses (Klein & Sato, 2000a,b). Polymorphism in MHC loci is selectively maintained, and there is evidence that infectious diseases play an important role in this selection (Hughes & Nei, 1988). Evolutionary mechanisms, such as the heterozygous advantage (Doherty & Zinkernagel, 1975) and frequency‐dependent selection (Takahata & Nei, 1990), are pathogen‐driven, maintaining high variation in MHC loci. Pathogen‐driven selection operates when specific alleles confer enhanced protection or susceptibility to a pathogen (Hughes & Nei, 1988; Jeffery & Bangham, 2000; Meyer & Thompson, 2001; Paterson, Wilson, & Pemberton, 1998). Furthermore, preservation of MHC allelic variability occurs by balancing selection due to two possible mechanisms: selection for resistance of pathogens or sexual selection via mate choice (Bernatchez & Landry, 2003; Knafler, Clark, Boersma, & Bouzat, 2012; Piertney & Oliver, 2006). Distinct populations are under different selective pressures; consequently, an allele beneficial in a given population may not be in another due to local adaptation to parasites or environmental conditions (Bateson, Whittingham, Johnson, & Dunn, 2015). On the other hand, lack of local adaptation may be observed when selection is weak, similar alleles are selected among populations, or the effect of genetic drift is strong (Kawecki & Ebert, 2004). Moreover, gene flow can limit local adaptation homogenizing genetic diversity among populations. Therefore, the distinct patterns observed across populations depend on the local and global relative effects of the selection, drift, and also gene flow (Bryja, Charbonnel, Berthier, Galan, & Cosson, 2007).

Two classes of MHC genes, MHC class I and MHC class II, are described for birds. MHC class I deals predominantly with intracellular infections (e.g., viruses) and MHC class II mostly with extracellular infections (e.g., bacteria) (Abbas, Lichtman, & Pober, 1994; Hughes & Yeager, 1998). There are also pathogens that activate both MHC classes I and II because they act intra‐ and extracellularly (Westerdahl, 2007). In the MHC class I genes, higher diversity is found in the exon 3, which encodes the domain that binds and presents peptides from pathogens triggering immune reactions. Nevertheless, polymorphism of the MHC class II DRB‐like genes for birds is higher in the exon 2 with a high rate of nonsynonymous substitutions (Kikkawa et al., 2009). The large nonsynonymous substitution rates that were expected under neutrality at exon 2 of the *Spheniscus* DRB1 gene is consistent with strong selection forces acting on the genetic region of antigen presentation in order to cope with various infections and parasites (Bollmer et al., 2007; Kikkawa et al., 2009). Therefore, different arrays of pathogens may generate different patterns of signature of selection between MHC I and II across populations of a species.

As mentioned above, MHC genes include the most polymorphic regions in vertebrates with high levels of heterozygosity (Janeway, Travers, Walport, & Shlomchik, 2004; Zagalska‐Neubauer et al., 2010). High polymorphism described in MHC is maintained by selection for pathogen resistance, and might even be aided by mate selection as was evidenced in Magellanic penguins (*Spheniscus magellanicus*; Knafler et al., 2012). Multiple loci of MHC can be detected in an individual (Zagalska‐Neubauer et al., 2010); therefore, this restricts the overall understanding of different alleles from each gene. Different situations can occur: In some species, genes within each class are both structurally and functionally divergent; however, in other groups, multiple loci may contain similar (and thus presumably more or less functionally equivalent) alleles, or identical alleles may even be shared among loci (Woelfing, Traulsen, Milinski, & Boehm, 2009). A MHC allele balance has to be maintained because a small amount of alleles in an individual can diminish its capacity to protect itself from pathogens, but an increased amount of alleles could invoke an autoimmune response in the individual; therefore, selection may favor individuals with an intermediate number of alleles (Milinski, 2006; Nowak, Tarczy‐Hornoch, & Austyn, 1992; Woelfing et al., 2009). The variability and complexity of the MHC genes vary between bird taxonomic groups: Some taxa, such as Passerines, have extremely complex MHC genes with high amounts of loci, alleles, and pseudogenes, while other species such as chickens or parrots have small compact MHC (Zagalska‐Neubauer et al., 2010). Interestingly enough, the MHC genes of Passerine birds tend to mirror their phylogeny (Zagalska‐Neubauer et al., 2010). The diversity of the avian MHC II gene sequences has been extensively studied in hens, quails, and passeriforms, but have been poorly studied in oceanic and coastal birds, including penguins (Kikkawa et al., 2005). Sequences of MHC II exon 2 have been compared between penguin species, including both Magellanic and Humboldt penguins, using traditional Sanger sequencing techniques (Kikkawa et al., 2005, 2009; Knafler et al., 2012; Tsuda et al., 2001); however, the complexity of these genes has not been evaluated for these species within and between populations. In the case of MHC class I, gene diversity has been recently evaluated for passeriform birds (Alcaide, Liu, & Edwards, 2013) but is unknown for penguins.

All extant penguins are part of the Spheniscidae family, the only family within the Sphenisciformes order. The *Spheniscus* genus includes four temperate penguins (*Spheniscus humboldti, S. mendiculus, S. demersus,* and *S. magellanicus*), resulting from a relatively recent speciation process (1.9 Mya; Subramanian et al. 2013). In South America, two of the four species are widely distributed across different latitudes: the Humboldt penguin (*S. humboldti*) and the Magellanic penguin (S. *magellanicus*). The Humboldt penguin breeding range is restricted to the Humboldt current and extends from Foca Island (5°S) in northern Peru to Metalqui (42°S) on the southern Pacific coast of Chile (De la Puente et al., 2013; Hiriart‐Bertrand et al., 2010; Paredes, Zavalaga, Battistini, Majluf, & Mc Gill, 2003; Vianna et al., 2014). Magellanic penguins' breeding range extends from 41°S on the eastern coast of South America, south around Cape Horn and north to 40°S on the Pacific coast, and includes the Malvinas–Falkland Islands (Boersma et al., 2013). Both species are colonial breeding seabirds, and adults show strong colony and nest philopatry (Araya et al., 2000; Simeone & Wallace, 2014; Teare et al., 1998). However, this behavior does not completely agree with population genetics results. A study between four colonies of Humboldt penguins in Peru and Chile using five microsatellite loci revealed a lack or reduced population structure (*F*_ST_ = 0–0.01, *p* < 0.05; Schlosser et al., 2009). The authors also describe high levels of heterozygosity (0.69–0.74) in all four locations, indicating that while some of the colonies have decreased in size and the species is declining (Zavalaga & Paredes, 1997), the current population is still large enough to maintain a high genetic diversity. Similarly, high heterozygosity (0.59) and low population structure (*F*_ST_ = 0.01) were found for the Magellanic penguins from six colonies in the southern Atlantic using four microsatellite loci and mtDNA COI sequences (*F*_ST_ = 0.08) (Bouzat, Walker, & Boersma, 2009). Contrarily to the latter results, Akst, Boersma, and Fleischer (2002) found a low heterozygosity level (0.03) in five microsatellite loci for the Galapagos penguin (*S. mendiculus*). Likewise, low nucleotide diversity (0.01) of the DRB1 gene of the MHC II was observed for Galapagos penguins, mainly when compared to the Humboldt penguin diversity (0.06; Bollmer et al., 2007). The low diversity was explained by the founder effect of the Galapagos species and the recurrent bottleneck effect due to the El Niño Southern Oscillation (ENSO) (Bollmer et al., 2007). However, the EN events may have impacted the northern populations of Humboldt penguins which should be further investigated. Likewise, during the last glacial maximum (LGM), the ice sheet covered the entire south of Chile from 56°S up to 42°S latitude (Clapperton, 1993; McCulloch et al., 2000). Ice sheet cover may have impacted the distribution of genetic diversity of Humboldt and Magellanic penguins along this region. Therefore, the extensive distribution of Humboldt and Magellanic penguins along the Pacific and Atlantic coasts and their environmental heterogeneity (e.g., ENSO, glaciation, temperature) may have implications in pathogen array and MHC I and II patterns of selection signature.

In this study, we evaluate the MHC I and MHC II adaptive gene diversity using 454 next‐generation sequencing for both Humboldt and Magellanic wild penguin colonies in Peru, Chile, and Argentina. We hypothesize that high gene flow among populations of Humboldt and Magellanic penguins limits local adaptation in the adaptive genes (MHC I and MHC II). Secondly, signatures of selection differ between MHC I and II, locations, and penguin species. Therefore, we evaluate the roll of drift, gene flow, and selection driving the MHC diversity in penguin populations of the Humboldt and Magellanic species. We also uncover the possible number of loci for MHC I and II for penguins and whether alleles are shared between species. Finally, we hypothesize that alleles from different locations and species are shared across phylogenetic clades due to the high gene flow among populations and also due to the recent speciation process within the *Spheniscus* genus.

## 2 Methods

### 2.1 Sample collection and DNA isolation

Samples were collected during penguin breeding seasons from the years 2010 to 2013. A total of 100 adult Humboldt penguins and 75 adult Magellanic penguins from seven breeding colonies in Peru, Chile, and Argentina (Table 1) were sampled. Penguins were quietly approached; a noose pole 1.5 m in length was used to lead the penguins out of their nests, and they were captured manually (Penguin Taxon Advisory Group, 2005). Penguins were handled following the standard methods described by the CCAMLR Ecosystem Monitoring Program (2004). Blood (1 cc) samples were obtained from the internal metatarsal vein or the brachial vein using a 23‐G needle and 3‐ml syringe and stored in 96% sterile ethanol for genetic analysis. To avoid resampling, penguins were marked temporarily with water‐resistant color markers. The bioethics permit was provided by the Pontificia Universidad Católica de Chile following CONICYT Bioethics Guidelines.

**Table 1:** Sampled breeding colonies of *Spheniscus* penguins, with the number of samples obtained for each colony, and the color code that is used for each barcode

	Locality	Latitude (S)	Longitude (W)	Country	Species	*N*	Color code
Barcode 1	Punta San Juan	15°22′	75°12′	Peru	*S. humboldti*	25	Blue
Barcode 2	Pan de Azucar Island	26°09′	70°41′	Chile	*S. humboldti*	25	Green
Barcode 3	Pajaro Island	29°35′	71°32′	Chile	*S. humboldti*	25	Yellow
Barcode 4	Cachagua Island	32°35′	71°27′	Chile	*S. humboldti*	25	Orange
Barcode 5	Puñihuil Island	41°55′	74°02′	Chile	*S. magellanicus*	25	Red
Barcode 6	Magdalena Island	52°55′	70°34′	Chile	*S. magellanicus*	25	Purple
Barcode 7	Puerto Deseado	47°54′	65°42′	Argentina	*S. magellanicus*	25	Pink

DNA extraction used a simple salt method with ammonium acetate 9M from Aljanabi and Martinez (1997). DNA quality and quantity (ng/μL) were estimated using a microplate reader (Epoch Microplate Spectrophotometer, BioTek, USA).

### 2.2 NGS Barcode preparation

One hundred Humboldt penguins (25 individuals per barcode from different localities) and 75 Magellanic penguins (25 individuals per barcode from different localities) were analyzed (Table 1). For MHC II exon 2, amplification of each individual was performed separately using the primers Lpen.hum1F and Lpen.hum2R which are located in the Intron 1 and Intron 2, respectively, and therefore can be used to amplify the complete exon 2 (Kikkawa et al., 2005). PCR amplification for MHC II exon 2 comprised an initial denaturalization step at 95°C for 10 min, followed by 35 cycles of 96°C for 30 s, and annealing temperature of 60°C for 30 s, and 72°C for two minutes. This was followed by a final extension at 72°C for four minutes. PCRs were performed in a total volume of 30 μl containing 2 μl of the template DNA, 1× reaction buffer, 1.5 mM of MgCl_2_, 200 μM of each dNTP, 0.5 μM of each primer, and 1 unit of Taq DNA polymerase Platinum (Invitrogen^®^, Sao Paulo, SP, Brazil). For MHC I exon 3, primers were designed based on Adelie penguin genome (*Pygoscelis adeliae*) (Zhang et al., 2014) where the gene was identified by homology from *Passer domesticus* MHC I exon 3 sequence (Genbank accession number: AY285003.1 from Bonneaud et al., 2004). The primers MHCIex3Pyg_2F (5′‐GTCTCCCTGGTCCTGTTTCA‐3′) and MHCIex3Pyg_2R (5′‐CGGCAGTACAAGGTCAGGAT‐3′) were designed using Primer‐BLAST tool in NCBI (http://www.ncbi.nlm.nih.gov/tools/primer-blast/). PCR amplification for MHC I exon 3 comprised an initial denaturing step at 95°C for 10 min, followed by 35 cycles of 96°C for 30 s, and annealing temperature of 55°C for 30 s, and 72°C for two minutes. The annealing temperature was chosen by a gradient of temperatures ranging from 50°C to 60°C with a 2°C difference between samples. PCR conditions were the same as used for MHC II exon 2. Amplification was visualized by electrophoresis in an agarose gel stained with gel red with a known concentration ladder (Lambda Hind III). PCR product quantification was performed measuring the pixels of the bands compared to the known ladder using image J software. For the MHC II sequences, we calculated the amount of GCTA concentration in femtomoles for each sequence using the known Humboldt penguin sequence (Kikkawa et al., 2005). However, for MHC I, this amount was considered at 50% because we did not have the complete sequence of the exon 3 plus the flanking regions (introns) due to incomplete *Pygoscelis* genome. All individual samples were included in each barcode Eppendorf tube with the same concentration until reaching a total of 1 ml. Seven barcodes were prepared to be analyzed with next‐generation sequencing (Roche/454 Life Sciences). Each barcode was composed of PCR products of MHC I exon 3 and MHC II exon 2 amplifications of the grouped individuals. Barcodes one through four had PCR products of Humboldt penguins, while barcodes five through seven had Magellanic penguins.

Five hundred nanograms of each DNA sample was sheared by nebulization, purified with the MinElute PCR Purification Kit (Qiagen), and subsequently processed according to the GS Rapid Library Preparation Kit Method Manual (Roche/454 Life Sciences, Branford, USA). The quality of DNA library was assessed by chip electrophoresis in the Bioanalyzer 2100 System (Agilent Technologies, Branford, USA). DNA fragments were equimolarly pooled and then clonally amplified using the GS Junior Titanium emPCR Lib‐L Kit (Roche/454 Life Sciences). Sequencing was performed using the GS Junior pyrosequencing system according to the shotgun sequencing method (Roche/454 Life Sciences).

### 2.3 NGS bioinformatic

Data analysis was performed following the steps presented in Galan, Guivier, Caraux, Charbonnel, and Cosson (2010) with some minor modifications as follows (Table S1). To analyze the reads obtained with the 454 NGS, we began by eliminating all sequences with incomplete barcode information which in this case were reads that were under 180 base pairs using Sequencher v. 5.1. (Gene Codes, Ann Arbor, MI, USA). To continue removing reads with incomplete barcode information, the MHC I and MHC II sequences were separated using reference sequences; for MHC II, we used a *Spheniscus humboldti* DRB1 partial gene (Genbank accession number AB154399.1), while for MHC I, we used a *Vultur gryphus* MHC I exon 3 clone (Genbank accession number GU060474) (Alcaide, Cadahia, Lambertucci, & Negro, 2010; Kikkawa et al., 2005). All sequences that did not align with a minimum of 70% alignment to any of the two reference sequences were eliminated. We separated both MHC I and MHC II sequences into two different datasets and the sequences were aligned at 100% alignment with Sequencher v. 5.1 to evaluate the obtained variants (possible alleles). All variants that occurred only once in each barcode were then removed, to improve resolution when examining variants according to their frequencies and to eliminate sequencing error (Oomen, Gillett, & Kyle, 2013).

To evaluate sequence quality due to NGS Phred scores, all sequences under 80% quality were eliminated. Finally, all sequences that presented deletions that were not multiples of three and could be an amino acid insertion were eliminated from the analysis (Galan et al., 2010). All MHC alleles were deposited in Genbank: MHC I for Magellanic (KX020586–KX020699) and Humboldt penguin (KX020700–KX020827) and MHC II for Magellanic (KX020882–KX020957) and Humboldt penguin (KX020828–KX020881).

### 2.4 Genetic diversity, genetic drift, and natural selection analysis

For all seven barcodes/locations and for both MHC exons, we calculated genetic diversity indexes: number of alleles, allelic diversity (Hd), nucleotide diversity (π). We also evaluated the signature of selection using the neutrality test of Tajima's D, Fu's Fs, and calculating the synonymous rates (Ks), nonsynonymous rates (Ka), and the Ka/Ks (or ω) ratio using DNAsp v. 5.10.1 (Librado & Rozas, 2009).

To evaluate the effects of genetic drift over genetic diversity, we measured the correlation between genetic diversity and population size for MHC I and II, expecting a positive correlation in case of drift. To compare MHC I and II, we calculated the mean‐centered estimate of genetic diversity by dividing the mean number of allele/locus in a particular population by the mean number of allele from all populations of each species as described by Bateson et al. (2015). Population size estimates for Humboldt penguins were obtained from Vianna et al. (2014) and for Magellanic penguins from Boersma et al. (2013).

### 2.5 Population differentiation in immune genes

To understand the role of natural selection on distinct populations, we calculated the *F*_ST_ among locations for each species using Arlequin v. 3.5.1.2 (Excoffier & Lischer, 2010). To evaluate isolation by distance (IBD), we performed a Mantel test with 10,000 permutations to assess the correlation significance between the pairwise *F*_*st*_ and geographic distances among locations. A weak or lack of IBD for one marker suggests high gene flow or selection favoring similar alleles in all locations. On the other hand, a strong pattern of IBD suggests a correlation between geographic distance and selection. Moreover, in the case the locations differ in pathogen pressure, virus, or bacteria, we should observe a different pattern of IBD between MHC I (intracellular infections) and MHC II (extracellular infections).

### 2.6 Phylogeny reconstruction

To evaluate the MHC alleles' relationship across populations in a spatial context but also between species, we used phylogenetic reconstruction. Independent barcode alleles were obtained using DNAsp v. 5.10.1 (Librado & Rozas, 2009), and then, they were joined together in a fasta file, with codes that specified the barcode/locality each sequence belonged to. We used Bayesian phylogenetic reconstruction to estimate the evolutionary relationship between MHC I and MHC II reads obtained for all seven barcodes. As an outgroup for MHC I exon 3, we used a *Vultur gryphus* MHC I exon 3 clone (Genbank accession number GU060474) (Alcaide et al., 2010), while for MHC II exon 2, we used an *Eudyptula minor* sequence (Genbank accession number AB302091.1), because it is the sister genus of *Spheniscus* (Baker, Pereira, Haddrath, & Edge, 2006). The best substitution model suitable for Bayesian phylogenetic reconstruction, selected by the Akaike information criterion (AIC) and Bayesian information criterion (BIC) using J‐ModelTest v.2.1.4 program (Posada, 2008), was GTR+G for MHC I and HKY + G for MHC II. The Bayesian analysis was run using MrBayes v.3.3 (Huelsenbeck & Ronquist, 2001). For the MHC I exon 3 phylogenetic analysis, four independent Markov chains, each beginning with a random tree, were run for 20,020,000 generations until reaching a split value of 0.009. While for the MHC II exon 2 phylogenetic analysis, four independent Markov chains, each beginning with a random tree, were run for 60,120,000 generations until reaching a split value of 0.009. To visualize the consensus trees, we used FigTree v.1.4.0 (Rambaut, 2009).

## 3 Results

Individuals were grouped by locality; therefore, seven barcodes were analyzed (four for Humboldt penguins and three for Magellanic penguins) with a maximum of 12,436 reads for barcode number five. In total, 64,171 reads were obtained (Table S1). After rigorous bioinformatic cleaning, only 8,527 reads were adequate for further analysis; this comprised 13.1% of all the original reads.

### 3.1 MHC diversity

**Figure 1:**
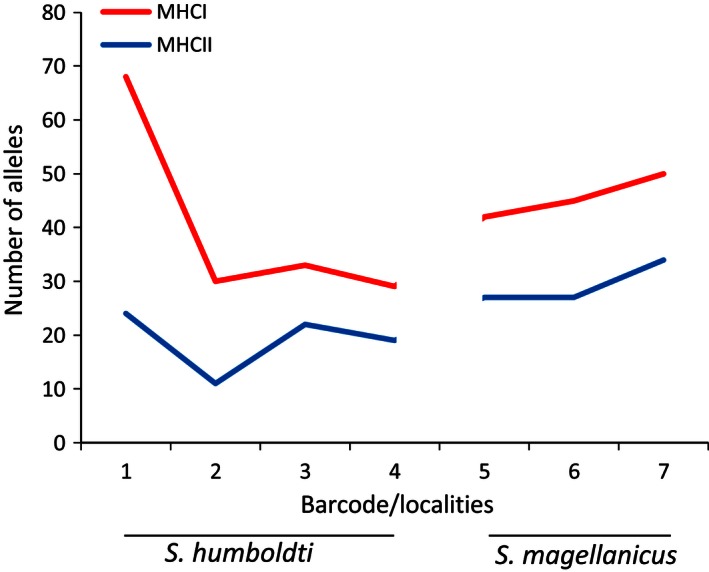
Amount of MHC I and MHC II alleles per localities (1–4 Humboldt penguins and 5–7 Magellanic penguins)

A total of 236 MHC I exon 3 alleles were identified for Humboldt and Magellanic penguin samples, with 38 alleles that were present in more than one locality. Of these 38 mixed locality alleles, six alleles are shared between Humboldt and Magellanic penguins. A total of 126 MHC II exon 2 alleles were found, with 17 mixed locality alleles and four that were present in both Humboldt and Magellanic penguins. Furthermore, a large amount of alleles were found for each locality with the most northernmost locality for Humboldt penguins (Barcode 1) having the highest allelic diversity with 68 different variants for MHC I (Table 2). The localities sampled from the extremes of the species distribution (Punta San Juan for Humboldt penguins and Puerto Deseado for Magellanic penguins) presented the highest number of alleles for both MHC I and MHC II genes. MHC II had a lower number of alleles compared to MHC I in all analyzed colonies (Figure 1). In the Punta San Juan Humboldt penguin colony, we evidenced 68 alleles for MHC I, present in 25 individuals, which averages 2.72 alleles per individual (Table 2, Figure 1), suggesting the presence of more than one loci.

**Table 2:** MHC I and MHC II reads, polymorphic sites (S), average of alleles per individual (No. of *A*/N), allelic diversity (Hd), nucleotide diversity (π), Tajima's *D*, Fu's *F*S, and nonsynonymous/synonymous substitution ratio (Ka/Ks) per barcode for both Humboldt and Magellanic penguins *significant values for Fu's Fs (p<0.03)

		*S. humboldti*				*S. magellanicus*		
		Barcode 1	Barcode 2	Barcode 3	Barcode 4	Barcode 5	Barcode 6	Barcode 7
MHCI	*N*	25	25	25	25	25	25	25
Exon 3	Reads	1,164	384	742	718	710	920	596
261 bp	S	77	50	47	49	53	63	57
	No. of alleles	68	30	33	29	42	45	50
	No. of A/N	2.72	1.2	1.32	1.16	1.68	1.8	2
	Hd	0.737	0.823	0.811	0.794	0.926	0.935	0.946
	π	0.031	0.040	0.033	0.044	0.048	0.060	0.050
	Tajima's *D*	−0.95	0.490	0.377	1.243	1.444	1.593	1.005
	Fu's *Fs*	−18.15*	0.606	0.109	4.643*	0.412	2.859*	−2.423
	Ka/Ks	3.205	2.787	2.814	3.298	3.949	2.632	3.942
	Ka	0.038	0.048	0.039	0.054	0.060	0.071	0.062
	Ks	0.012	0.019	0.015	0.019	0.018	0.040	0.019
MHCII	N	25	25	25	25	25	25	25
Exon 2	Reads	524	254	580	458	698	312	468
270 bp	S	38	31	38	50	46	47	46
	No. of alleles	24	11	22	19	27	27	34
	No. of A/N	0.96	0.44	0.88	0.76	1.08	1.08	1.36
	Hd	0.794	0.780	0.693	0.705	0.404	0.915	0.877
	π	0.034	0.034	0.031	0.036	0.018	0.047	0.492
	Tajima's *D*	0.891	1.543	0.825	0.331	−1.193	1.084	1.589
	Fu's *F*s	3.201*	10.652*	3.598*	6.720*	−1.967*	2.711*	1.929*
	Ka/Ks	0.817	0.952	0.868	0.879	0.930	1.004	1.312
	Ka	0.032	0.034	0.030	0.036	0.183	0.046	0.051
	Ks	0.045	0.040	0.038	0.043	0.022	0.058	0.050

**Figure 2:**
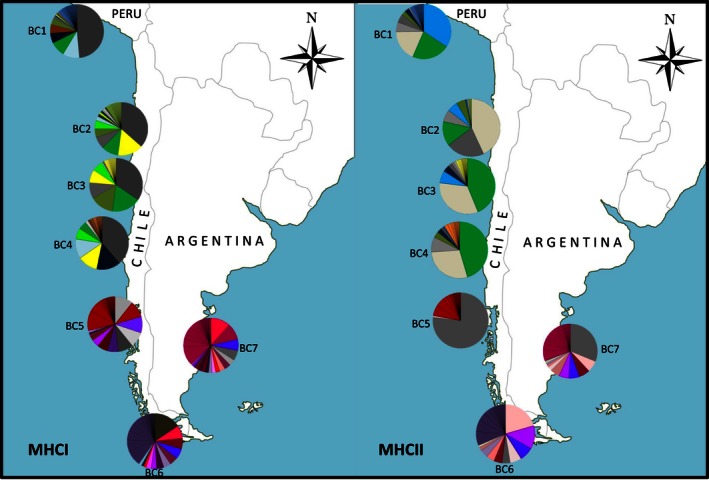
Allelic frequency pie charts per locality sampled. Unique alleles are not separated by color in each barcode; however, they are separated by black lines. Humboldt penguin colonies correspond to Barcode 1 (blue), Barcode 2 (green), Barcode 3 (yellow), and Barcode 4 (orange). Magellanic penguin colonies correspond to Barcode 5 (red), Barcode 6 (purple), and Barcode 7 (pink)

For MHC I and MHC II, Magellanic penguins presented more homogeneous allelic frequencies than Humboldt penguins, where one or two alleles were usually dominant in a population (Figures 2, S1, S2); however, Humboldt penguins presented a larger amount of alleles compared to Magellanic penguins (Figure 1). The frequency of the dominant allele for Humboldt penguins ranged from 0.35 to 0.49 for MHC I and from 0.34 to 0.45 for MHC II (Figure S1). For MHC I in Humboldt penguins, the same allele was the most frequent in all four localities (dark gray in Figure 2). On the other hand, the most frequent allele for Magellanic penguin ranged from 0.12 to 0.16 for MHC I and from 0.21 to 0.77 for MHC II. For both MHC I and MHC II, the two barcodes with the largest frequency of private alleles were Magdalena Island and Puerto Deseado Magellanic penguin colonies. The exception to this rule is the locality of Puñihuil Island (Barcode 5) for Magellanic penguin where a dominant allele and low genetic diversity were found (Figures 2, S2). Although Humboldt penguin colonies present a large number of private alleles for MHC I and MHC II, the most frequent alleles were shared between locations (Figures 2, S1). The higher number of alleles was observed in lower latitudes for both genes and species, with higher number of alleles in Punta San Juan for Humboldt penguin (68 alleles, S = 77, for MHC I and 24 alleles, *S* = 38 for MHC II) and Puerto Deseado for Magellanic penguin (50 alleles, *S* = 57 for MHC I and 34 alleles, *S* = 46 for MHC II) (Table 2). Furthermore, MHC I always presented a greater number of alleles per locality than MHC II for both species, which can be explained by the presence of more than one locus (Figures 1 and 2).

The MHC I allelic and nucleotide diversities for each population were high for most of the localities, ranging from Hd = 0.737 to 0.823 and π = 0.031 to 0.044 for the Humboldt penguin and from Hd = 0.926 to 0.946 and π = 0.048 to 0.060 for the Magellanic penguin (Table 2, Figure S3). Furthermore, high diversity for MHC II was found for all Humboldt penguin localities (Hd = 0.693–0.794; π = 0.031–0.036) and Magellanic penguin localities (Hd = 0.404–0.915, π = 0.018–0.049) with exception of Puñihuil Island that presented a very low diversity (Hd = 0.404, π = 0.018) (Table 2, Figure S3).

### 3.2 MHC selection and genetic drift

**Figure 3:**
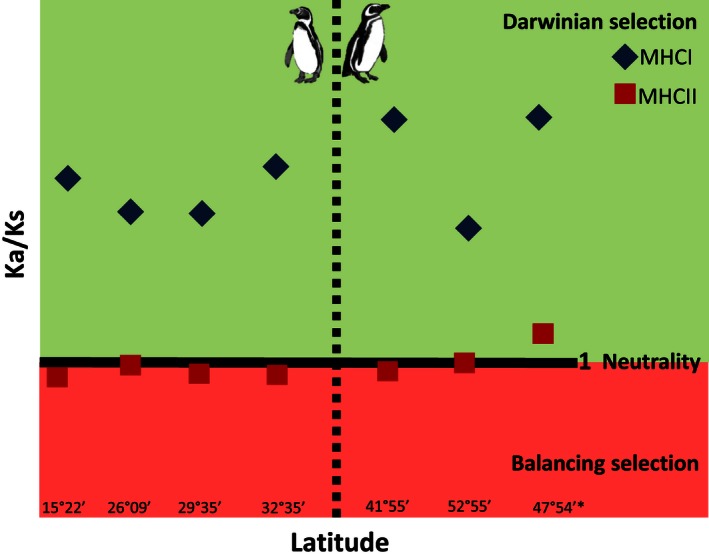
Synonymous (Ks)/nonsynonymous (Ka) substitution ratio for Humboldt and Magellanic penguin MHC I and MHC II genes (highest value observed 3.9). *X*‐axis corresponds to latitude of all localities (1–4 Humboldt penguins and 5–7 Magellanic penguins). *the only locality from the Atlantic coast

Marked differences between MHC I and MHC II Ka/Ks ratio values were found. All localities presented highly positive Ka/Ks ratio for MHC I exon 3 with values ranging between 2.6 and 3.9, while a balancing or neutral effect was found for all localities for MHC II exon 2 with values between 0.8 and 1.3 (Table 2, Figure 3). These results are consistent with Fu's *Fs* values which were mostly positive and significant for all localities and both species suggesting balancing selection over the MHC II with exception of Puñihuil Island (Table 2). MHC I Ka/Ks ratio was variable between localities and higher for the localities in the extremes of the distribution for each species. On the other hand, for MHC II, the Ka/Ks ratio was similar between localities.

We did not observe an overall increment of genetic diversity with the increase in population size for both genes and species (Figures S4 and S5), thus suggesting a weak genetic drift effect over the genetic diversity or a strong selection.

### 3.3 MHC population differentiation and local adaptation

**Figure 4:**
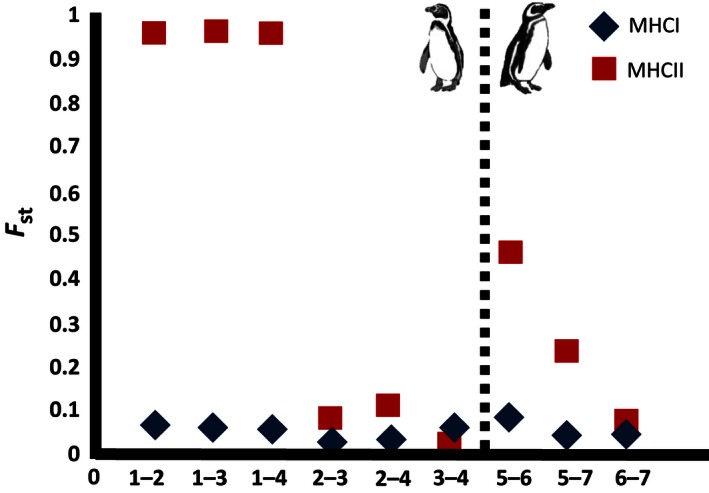
Population pairwise *F*_ST_ of Humboldt and Magellanic penguin colonies (barcode 1 to 7)

Higher significant population structure was detected for the MHC II of both Humboldt (*F*_ST_ = 0.023–0.959) and Magellanic penguins (*F*_ST_ = 0.070–0.457), compared to lower but significant *F*_*st*_ values found for MHC I of Humboldt (*F*_ST_ = 0.014–0.066) and Magellanic penguins (*F*_ST_ = 0.041–0.084; Table 3). The highest *F*_*st*_ values were observed for MHC II between the Punta San Juan colony against all other localities (*F*_ST_ = 0.9548–0.9585, Figure 4). Therefore, a significant pattern of isolation by distance (IBD) was observed only for Humboldt penguin MHC II (*Z *=* *4.87, *r *=* *0.93, *p *=* *.04; Table 4). These data suggest local adaptation mainly for the northernmost studied colonies of Punta San Juan for MHC II.

**Table 3:** *F*_ST_ values between locations (Barcode 1–7) for Humboldt (A) and Magellanic (B) penguins diagonal below MHC I, diagonal above MHC II

(A)				
	1	2	3	4
1	0	0.956	0.959	0.955
2	0.065	0	0.079	0.110
3	0.066	0.031	0	0.023
4	0.056	0.014	0.054	0

(B)				
	5	6	7	
5	0	0.457	0.228	
6	0.084	0	0.070	
7	0.041	0.051	0	

All values were significant (*p *<* *0.0001).

**Table 4:** Isolation by distance among *Spheniscus humboldti* and *S. magellanicus* populations. Results of Mantel test significant only for MHC II for *S. humboldti*

Marker	Species	*Z*	*r*	*p*
MHC I	*S. humboldti*	0.35	0.58	0.23
	*S. magellanicus*	0.22	−0.24	0.66
MHC II	*S. humboldti*	4.87	0.93	0.04^a^
	*S. magellanicus*	1.06	0.38	0.50

a Significant value (p< .05)

### 3.4 MHC lineages

**Figure 5:**
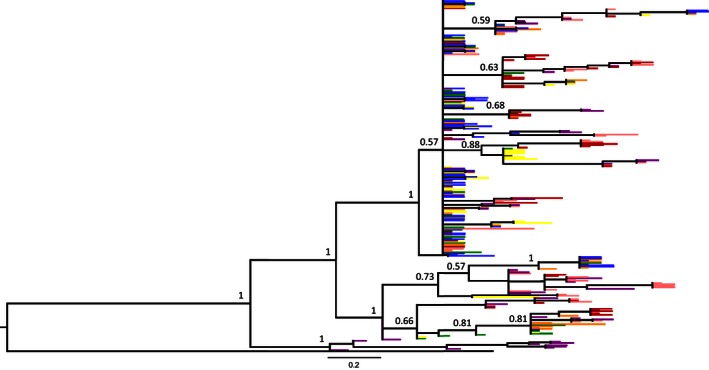
Bayesian phylogenetic reconstruction of complete MHC I exon 3 (261 bp) of four Humboldt and three Magellanic penguin colonies. Posterior support values are shown for each node >0.5. Humboldt penguin colonies correspond to Barcode 1 (blue), Barcode 2 (green), Barcode 3 (yellow), and Barcode 4 (orange). Magellanic penguin colonies correspond to Barcode 5 (red), Barcode 6 (purple), and Barcode 7 (pink)

**Figure 6:**
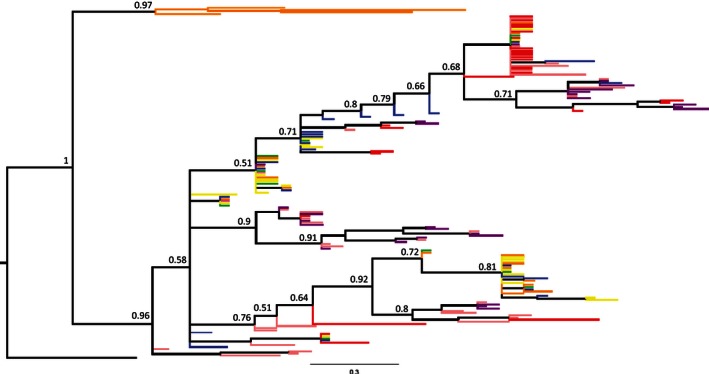
Bayesian phylogenetic reconstruction of complete MHC II exon 2 (270 bp) of four Humboldt and three Magellanic penguin colonies. Posterior support values are shown for each node >0.5. Humboldt penguin colonies correspond to Barcode 1 (blue), Barcode 2 (green), Barcode 3 (yellow), and Barcode 4 (orange). Magellanic penguin colonies correspond to Barcode 5 (red), Barcode 6 (purple), and Barcode 7 (pink)

The Bayesian phylogenetic reconstruction for both MHC I and MHC II alleles showed the absence of monophyly for both Humboldt and Magellanic penguins, and alleles were equally present in almost all clades (Figures 5 and 6). However, MHC I phylogeny showed three main clades and a polytomy in the most diverse clade (Figure 5). The only clade exclusive for one locality is from Magdalena Island (Barcode 6, posterior support value of 1). For MHC II, only two clades were monophyletic for each species: (1) Humboldt penguin (Cachagua, Barcode 4) with a posterior support value of 0.97 and (2) Magellanic penguin clade composed by alleles from all three localities (Figure 6). Furthermore, the phylogenetic reconstruction of MHC II exon 2 was more conserved than the MHC I and presented more specific lineages.

## 4 Discussion

Our study presents the MHC diversity of Humboldt and Magellanic penguins obtained from wild colonies in a large part of their reproductive distribution in Peru, Chile, and Argentina. Although previous studies were conducted with MHC II in penguin species (Kikkawa et al., 2005, 2009; Knafler et al., 2012; Tsuda et al., 2001), this is the first study where MHC I has been evaluated in these species.

### 4.1 Evidence of multigene MHC I loci

For some localities, such as Punta San Juan, we evidenced an average of 2.72 alleles per individual for MHC I, which can be explained by the presence of pseudogenes also described in a large number of studies for this gene (Cadavid, Hughes, & Watkins, 1996; Hess, Gasper, Hoekstra, Hill, & Edwards, 2000; Kryspin‐Sorensen, Johansen, & Kasten, 1991; Nadachowska‐Brzyska, Zielinski, Radwan, & Babik, 2012). The separation of genes from pseudogenes using this sequencing method is impossible; however, it has been demonstrated that these pseudogenes are functional, and therefore, this region has now been classified as a multigene family (Alcaide et al., 2013). Recently, between three and ten MHC sequences (variants/alleles) per individual were found when evaluating the MHC I exon 3 in songbirds (Alcaide et al., 2013); therefore, the 2.72 value that was found in this study could be categorized as low. Hence, in this study, we present evidence for multigene MHC I loci for at least one colony of Humboldt penguins (Barcode 1, Punta San Juan). On the other hand, MHC II did not present more than two alleles per individual, ranging from 0.44 to 1.36.

### 4.2 MHC I and MHC II polymorphism

The chicken MHC structure is simplified compared to mammals (Kaufman, Milne, & Goebel, 1999; Miller et al., 2004). However, more studies have been developed in nonmodel avian species and this has shown that the simple MHC found in Galliformes is not representative for all bird taxa, and there is an extreme amount of variation (Bollmer et al., 2007). Therefore, a large number of divergent alleles have been described for MHC genes in wild bird populations (Westerdahl, 2007; Westerdahl et al., 2005). We evidenced high MHC diversity in wild Humboldt and Magellanic penguins increasing the complexity of the analysis of these genes (especially MHC I) in avian populations. The MHC I exon 3 is more diverse than MHC II exon 2 and is extremely complex in *Spheniscus* penguins, including also interspecies alleles. Kikkawa et al. (2009) analyzed the MHC II of all four species that belong to the *Spheniscus* genus showing that both Humboldt and Magellanic penguins presented the greatest number of alleles, and most of the individuals were heterozygous, while homozygosity for the African and Galapagos penguins was higher at 75% and 83%, respectively. Unfortunately in our study, we were not able to evaluate heterozygosity due to the pooling of individuals per locality and barcode. However, this is the first study to describe the MHC I for penguins and its diversity compared to MHC II genes. In this case, we identified 236 alleles for MHC I and 126 alleles for MHC II when analyzing 175 individuals, which in the case of MHC II is similar to the number of alleles described for 20 Humboldt (nine alleles) and five Magellanic penguins (eight alleles) (Kikkawa et al., 2009).

Although MHC II showed lower diversity than MHC I, it still had high levels of diversity. Low MHC II exon 2 variations were found for the sister species of the Humboldt penguin, the Galapagos penguins (*Spheniscus mendiculus*), with only three alleles in 30 individuals; however, in this study, this analysis was performed with only partial allelic sequences from exon 2 (Bollmer et al., 2007). It is believed that the low MHC II allelic diversity found for Galapagos penguins might be due to genetic drift, given the species demographic history: with an initial founder effect and repeated consequent bottlenecks due to the increase in oceanic temperatures during El Niño events over thousands of years (Bollmer et al., 2007). Although El Niño events have caused mortality in Humboldt penguins in Peru (Vianna et al., 2014), a bottleneck effect was not detected in Punta San Juan using microsatellite markers (Schlosser et al., 2009). Likewise, high MHC diversity was observed for Humboldt penguin in this same locality.

Higher number of alleles (*A* = 45, *n* = 100) were described for MHC II *S. magellanicus* in a previous study carried out in Punta Tombo, Argentina (Knafler et al., 2012). These values were similar to our study, where we described a total of 126 alleles for *S. magellanicus* with higher numbers of alleles in Argentina (*A* = 34, *n* = 25, Puerto Deseado) compared to Chile (*A* = 27, *n* = 25, Puñihuil Island and Magdalena Island as well). Furthermore, Kikkawa et al. (2005) studied the MHC II gene of the Humboldt penguin describing high diversity of exon 2 with 27 polymorphic base pairs in 15 captive and five wild‐caught individuals. Likewise, we observed 31–50 polymorphic sites for exon 2 MHC II (270 bp) in 100 Humboldt penguins, while for Magellanic penguins, it ranged between 46 and 47 sites. The most frequent alleles correspond to shared alleles between localities, which is usually the case in species that have a metapopulation organizational structure. The same pattern was described for other species that are distributed in metapopulations (e.g., Boyce et al., 1997; Oliver, Lambin, Cornulier, & Piertney, 2009). Bouzat et al. (2009) using nuclear microsatellites and mitochondrial DNA markers suggest a metapopulation dynamic for Magellanic penguins, as well as Schlosser et al. (2009) for Humboldt penguins. Our findings of MHC I and MHC II allele frequency support this theory for both Humboldt and Magellanic penguins, with a reduced population structure and a lack of isolation by distance detected for both species.

### 4.3 Evolutionary forces acting over MHC I and MHC II

The effects of genetic drift over genetic diversity are weak in the overall populations of Humboldt and Magellanic penguins, but gene flow and selection are important evolutionary forces. Evidence of positive selection was detected for MHC I exon 3, with Ka/Ks ratio values ranging from 2.6 to 3.9 for Humboldt and Magellanic penguins. However, these data should be considered with caution due to more than one loci detected for this marker (e.g., pseudogene or function loci). Positive selection was detected only for MHC I, while for MHC II, where most Ka/Ks values were <1 for both species suggesting a purifying, stabilizing, or balancing selection. However, all of the colonies presented values very close to 1 which implies a neutral selection. The balancing selection for MHC II was also supported by Fu's Fs positive and significant values. Studies have also evidenced balancing selection for MHC II; one example of this would be a metapopulation of water voles in Scotland (Oliver et al., 2009). Furthermore in this metapopulation of water voles, a balancing selection varied in time, but not spatially. The authors attribute this to a possible selection that is being aided by temporal changes in the abundance or diversity of parasites in the metapopulation. A balancing selection in MHC II as was evidenced with Humboldt and Magellanic penguins may be attributed to pathogen pressures (Meyer & Thompson, 2001) and mate choice selection (Penn & Potts, 1999) on the one hand and the increase in autoimmunological diseases that could arise due to the decrease in self‐recognition that can be affected on the other hand (Tizard, 2009). Likewise, Knafler et al. (2012) found evidence of positive selection (Ka/Ks = 7.01) for MHC II in Magellanic penguins in Punta Tombo, Argentina, rather than a balancing selection as we evidenced in this study. Furthermore, Kikkawa et al. (2009) also found strong positive selection (11 for Humboldt and 12 for Magellanic penguins) for MHC II exon 2 for the *Spheniscus* genus and attribute this to strong positive forces acting on the genetic region of antigen presentation in order to cope with various infections. MHC I presents intracellular foreign peptides (for example viruses), while MHC II presents extracellular foreign peptides (for example bacteria) to T‐cell receptors (Gómez‐Lucía, 2007). Therefore, a higher MHC I gene diversity could probably mean a higher protection against a larger diversity of viruses or it could just mean that due to the higher diversity of viruses compared to bacteria, avian hosts have a higher diversity of MHC I than MHC II.

Moreover, selection may vary spatially among Humboldt and Magellanic penguin populations. Punta San Juan showed high Ka/Ks ratio, specially for MHC I, and the highest number of MHC I and MHC II alleles for all Humboldt penguin colonies. The number of alleles for MHC I in Punta San Juan was over twofold the number of alleles in all other localities. A significant pattern of isolation by distance (IBD) identified for MHC II for the Humboldt penguin suggests local adaptation. This pattern is explained mainly by the high significant values of *F*_*st*_ between Punta San Juan and the other locations. This might be due to the higher immunological pressure that this colony might be receiving because it is the locality that is at the lowest latitude, and a higher diversity of pathogens is associated with lower latitudes (latitudinal diversity gradient hypothesis) (Hawkins, Cornell, & Hochberg, 1997; Schemske, Mittelbach, Cornell, Sobel, & Roy, 2009). Furthermore, in a recent study of Humboldt and Magellanic penguins, avian malaria was diagnosed in three penguins only in the Punta San Juan Humboldt penguin colony, while all other colonies further south were negative for parasitemia of avian malaria (Sallaberry‐Pincheira et al., 2015). Further epidemiological studies have to be conducted in wild penguin colonies to increase knowledge about this hypothesis. High gene flow described for both species (Bouzat et al., 2009; Schlosser et al., 2009) may homogenize the adaptive genetic diversity between localities, limiting local adaptation. However, in some localities at the extreme of the distribution such as Punta San Juan, the gene flow is more restricted, as described using microsatellite data (Schlosser et al., 2009), promoting local adaptation observed here for MHC.

### 4.4 Trans‐species alleles in both MHC I and MHC II

Remarkably, of the 236 alleles described here for MHC I, six were shared between Humboldt and Magellanic penguins, while for MHC II, four mixed alleles were identified. In a study carried out on Galapagos penguins involving 157 bp of the MHC II exon 2, one allele was found to be identical between Humboldt and Magellanic penguins, which was not expected because Galapagos penguins are currently considered the sister species of Humboldt penguins, and Magellanic penguins are considered the sister species of African penguins (Bollmer et al., 2007). As suggested by the authors in this study, we increased the number of analyzed individuals and a greater amount of interspecific alleles were found. Kikkawa et al. (2009) determined that the allele sharing in the *Spheniscus* genus emphasizes that they are closely related species with an allopatric distribution and that some common alleles have underdone trans‐species inheritance probably because of balancing selection or rapid speciation processes. Furthermore, the low posterior support values obtained in our phylogenetic analysis and the increased amount of polytomies highlight the uncertainty of the branching nodal origins, as well as for the complete *Spheniscus* genus with much fewer sequences (Kikkawa et al., 2009). This allele sharing for MHC II exon 2 has also been documented in other species, such as primates and European Bison (Otting, de Groot, Doxiadis, & Bontrop, 2002; Radwan, Kawalko, Wojcik, & Babik, 2007), where recent speciation processes were attributed. We propose two possible explanations: (1) In fact, this is attributed to the *Spheniscus* genus recent speciation process approximately 1.9–4 mya (Baker et al., 2006; Subramanian et al. 2013) and even recent introgression described for Humboldt and Magellanic penguins in Puñihuil island (Barcode 5; Simeone et al., 2009) or (2) the antigen‐presenting region of the MHC needs to be so diverse that the DNA polymorphism is high and what we evidence is in fact homoplasy. However, it is difficult to distinguish which of the two explanations are acting on the trans‐species alleles evidenced in these species. Furthermore, the associated pattern of trans‐species polymorphism, in which similar or even identical alleles are shared among species, is often used to infer that balancing selection has occurred (e.g., Cutrera & Lacey, 2007). The authors evaluate MHC genes of 18 species of *Ctenomys* rodents suggesting historical balancing selection due to the presence of specific pathogens. Kikkawa et al. (2009) concludes that the trans‐species allele sharing is complex to evaluate asit is difficult to distinguish between the effects of balancing selection, gene conversion, and rapid speciation on allele retention between species.

### 4.5 Importance in conservation and species management

The depletion of variation at MHC loci, which play a crucial role in pathogen recognition, has been postulated to be one of the most important extinction risk factors for endangered populations (O'Brien & Evermann, 1988). Quickly evolving parasites, such as viruses, may adapt to the most common host genotypes and escape detection of their antigens by the host's adaptive immune system (Ejsmond & Radwan, 2011). The large amount of rare allelic variants described in our study could increase the immunological defense both penguin species have against parasites, because these are unlikely to adapt to new, less frequent variants (Borghans, Beltman, & De Boer, 2004). Furthermore, heterozygote advantage in resistance to parasites contributes to higher polymorphisms at MHC loci, and this allows a broader range of antigens to be presented (McClelland, Penn, & Potts, 2003). Although heterozygosity could not be evaluated, we observed high diversity for all populations for both species which allows more pathogenic antigens to be presented to T lymphocytes and thus offer a higher immunological defense. High MHC I and MHC II gene diversity described here in both Humboldt and Magellanic penguins is extremely advantageous for the survival of the species. Therefore, this diversity must be maintained by avoiding local population bottleneck in the wild, and to do so, conservation projects for both species have to be managed at a metapopulation level.

## Conflict of Interest

None declared.

## Data Accessibility

MHC sequences: GenBank accession numbers KX020586–KX020957.
