# Supplementary figures and images for: Contrasting patterns of selection between MHC I and II across populations of Humboldt and Magellanic penguins
Source: Ecol Evol. 2016 Sep 28;6(20):7498–510. doi: 10.1002/ece3.2502 (PMC5513272; doi:10.1002/ece3.2502)

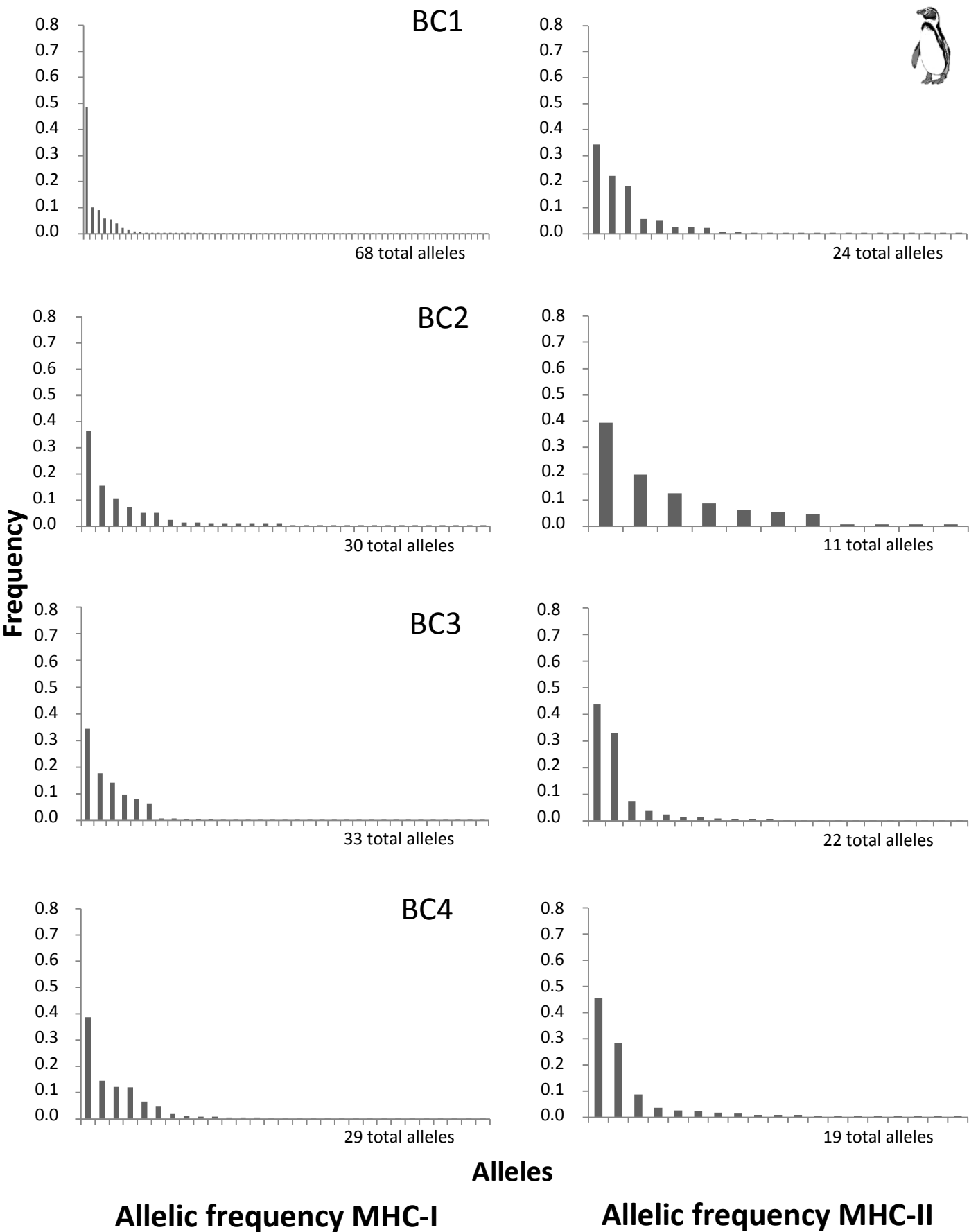

Supplement: Supplementary file 1 [file ECE3-6-7498-s001.pdf]

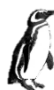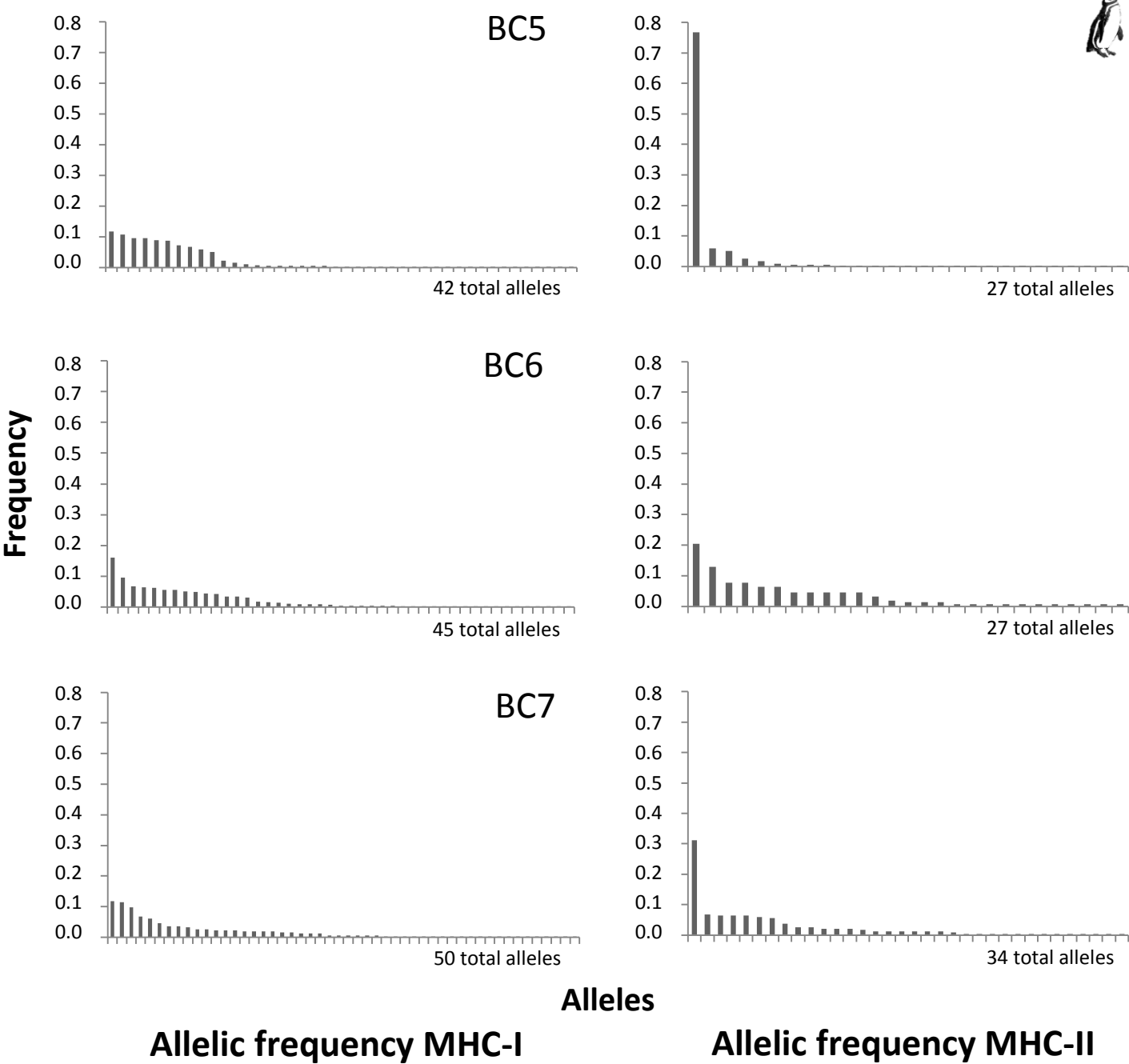

Supplement: Supplementary file 2 [file ECE3-6-7498-s002.pdf]

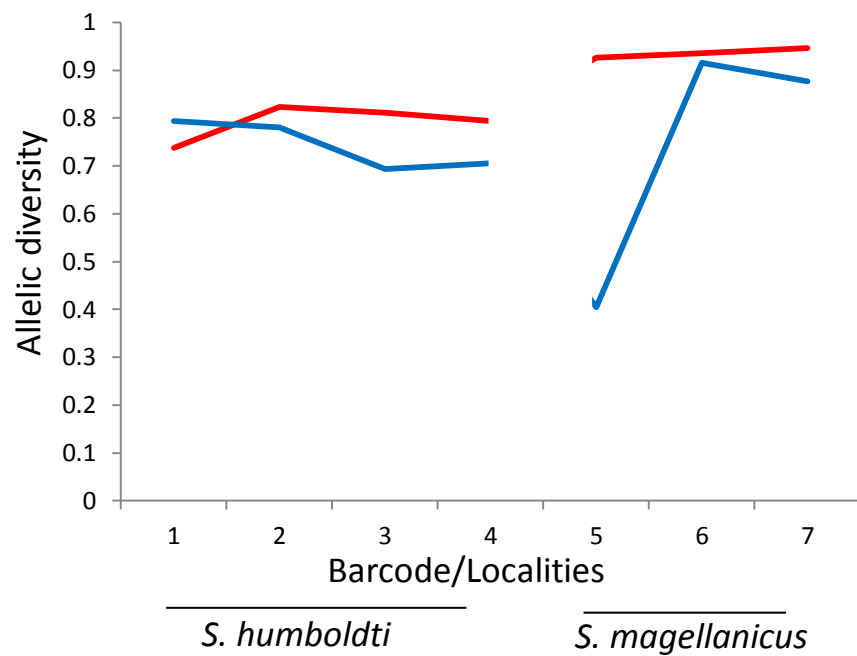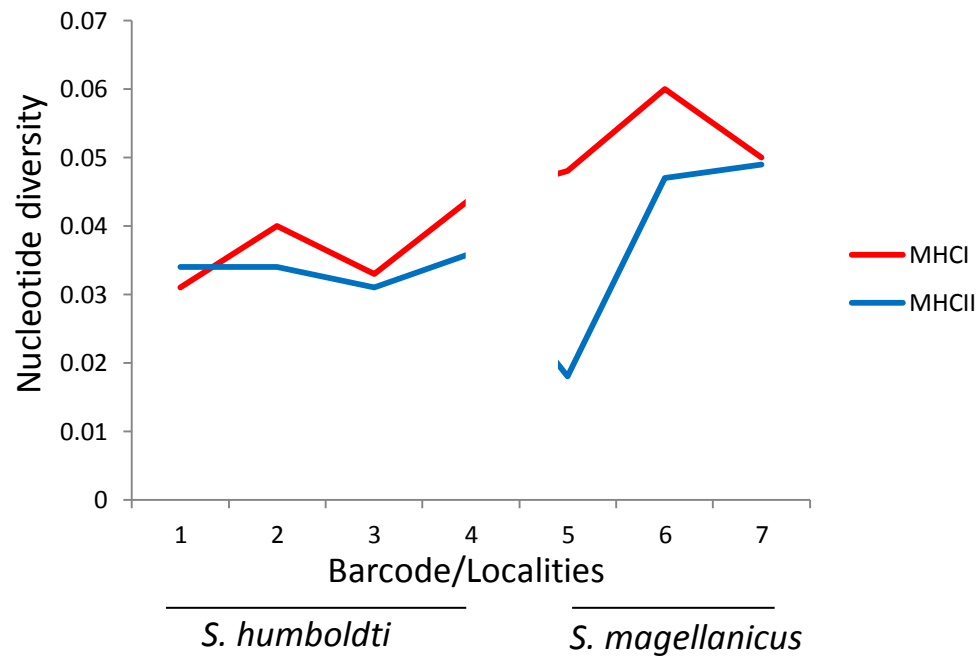

Supplement: Supplementary file 3 [file ECE3-6-7498-s003.pdf]

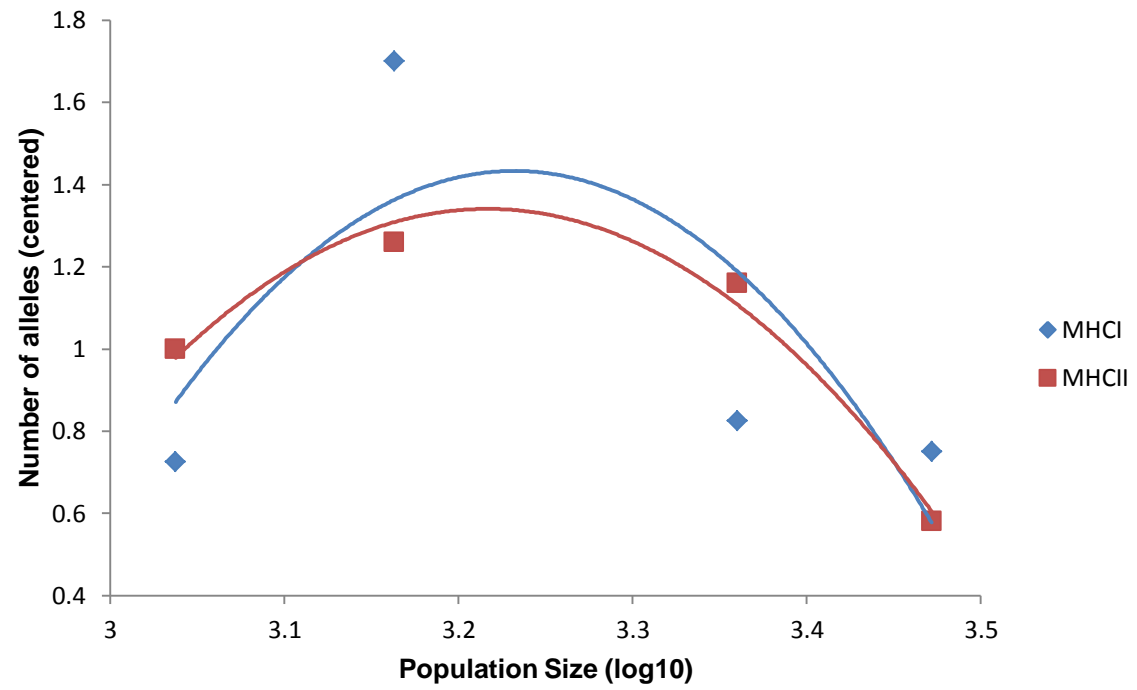

Supplement: Supplementary file 4 [file ECE3-6-7498-s004.pdf]

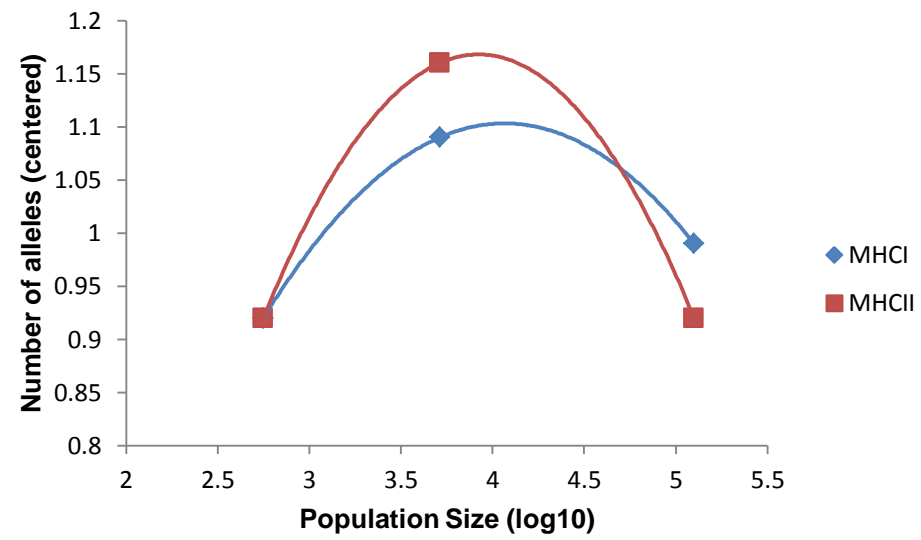

Supplement: Supplementary file 5 [file ECE3-6-7498-s005.pdf]
