# Supplementary material for: Contrasting patterns of selection between MHC I and II across populations of Humboldt and Magellanic penguins
Source: Ecol Evol. 2016 Sep 28;6(20):7498–510. doi: 10.1002/ece3.2502 (PMC5513272; doi:10.1002/ece3.2502)
Supplement: Supplementary file 6 [file ECE3-6-7498-s006.docx]

Table S1: Bioinformatic cleaning of MHCI and MHCII reads based on Galan *et al.* (2010)

|  |  | Barcodes | | | | | | | | | | | | | |  |
| --- | --- | --- | --- | --- | --- | --- | --- | --- | --- | --- | --- | --- | --- | --- | --- | --- |
|  |  | 1 |  | 2 |  | 3 |  | 4 |  | 5 |  | 6 |  | 7 |  | Total |
|  | Original | 8876 |  | 5185 |  | 7921 |  | 8734 |  | 12189 |  | 10936 |  | 10326 |  | 64168 |
|  | After eliminating al sequences shorter than 180 bp | 4487 |  | 2086 |  | 3607 |  | 3421 |  | 4710 |  | 5298 |  | 4441 |  | 28050 |
|  |  | MHCI | MHCII | MHCI | MHCII | MHCI | MHCII | MHCI | MHCII | MHCI | MHCII | MHCI | MHCII | MHCI | MHCII |  |
| Reads | After separation using a reference sequence | 2120 | 1644 | 759 | 876 | 1311 | 1480 | 1367 | 1470 | 1514 | 1808 | 2165 | 1778 | 1252 | 1873 | 21417 |
|  | After eliminanting single variants | 1287 | 638 | 411 | 276 | 806 | 650 | 801 | 606 | 805 | 776 | 986 | 370 | 636 | 581 | 9629 |
|  | After eliminating sequenceswith deletions | 1176 | 544 | 400 | 273 | 773 | 620 | 769 | 489 | 788 | 706 | 941 | 315 | 624 | 467 | 8885 |
|  | After eliminating all sequences that had a quality scores less than 80% | 1164 | 524 | 384 | 254 | 742 | 580 | 718 | 458 | 710 | 698 | 920 | 312 | 595 | 468 | 8527 |
|  |  |  |  |  |  |  |  |  |  |  |  |  |  |  |  |  |
