# Supplementary material for: Contrasting patterns of selection between MHC I and II across populations of Humboldt and Magellanic penguins
Source: Ecol Evol. 2016 Sep 28;6(20):7498–510. doi: 10.1002/ece3.2502 (PMC5513272; doi:10.1002/ece3.2502)
Supplement: Supplementary file 7 [file ECE3-6-7498-s007.docx]

**Supplementary material legend**

Table S1: Bioinformatic cleaning of MHCI and MHCII reads based on Galan et al. (2010)

Fig. S1: Allele frequency for MHCI and MHCII Humboldt penguin genes in four colonies (BC1=Punta San Juan; BC2=Pan de Azucar Island; BC3=Pajaros Island; BC4=Cachagua Island).

Fig. S2: Allele frequency for MHCI and MHCII Magellanic penguin genes in three colonies (BC5=Puñihuil Island; BC6=Magdalena Island; BC7=Puerto Deseado).

Fig. S3: Allelic diversity (*h*) and nucelotide diversity (π) of Humboldt and Magellanic penguin MHCI and MHCII genes for each localities (1-4 Humboldt penguins and 5-7 Magellanic penguins).

Fig. S4 – Number of alleles for MHCI and MHCII related to population size (log 10-transformed) across four populations of Humboldt penguin.

Fig. S5- Number of alleles for MHCI and MHCII related to population size (log 10-transformed) across four populations of Magellanic penguins.
